# Lactylation in cancer: Advances and opportunities for treatment resistance

**DOI:** 10.1002/ctm2.70478

**Published:** 2025-09-29

**Authors:** Keke Xu, Yiyi Shou, Ruiqi Liu, Hao Xiong, Xiaomeng Dai, Xuanwen Bao, Xiaoyan Chen, Luanluan Huang, Hailong Sheng, Haibo Zhang, Yanwei Lu

**Affiliations:** ^1^ Cancer Center Department of Radiation Oncology Zhejiang Provincial People's Hospital (Affiliated People's Hospital) Hangzhou Medical College Hangzhou Zhejiang China; ^2^ School of Basic Medical Sciences and Forensic Medicine Hangzhou Medical College Hangzhou Zhejiang China; ^3^ Department of Pathology Tongde Hospital of Zhejiang Province Hangzhou Zhejiang China; ^4^ Department of Medical Oncology The First Affiliated Hospital School of Medicine Zhejiang University Hangzhou Zhejiang China

**Keywords:** chemotherapy, immunotherapy, lactylation, targeted therapy, treatment resistance

## Abstract

**Background:**

Lactylation, a recently identified post‐translational modification that utilizes lactic acidas a substrate, has emerged as an important regulator of gene expression andprotein function. Since its discovery in 2019, lactylation has beenincreasingly recognized for its roles in cancer biology and treatment response.

**Main text:**

Lactylationis strongly associated with tumor progression and malignancy, underscoring itspotential as a therapeutic target. Recent studies also link lactylation tocancer treatment resistance, suggesting that modulating this modification couldenhance therapeutic efficacy. As treatment resistance remains a major clinicalchallenge in oncology, accumulating evidence indicates that dysregulatedlactylation contributes to resistance across chemotherapy, immunotherapy, targeted therapy, and radiotherapy. Preclinical and clinical research has begunto delineate the molecular pathways through which lactylation shapes theseresistance processes, and experimental approaches targeting lactylation arebeing explored to restore therapeutic sensitivity.

**Conclusion:**

This review systematically summarizes the mechanisms of lactylation and its roles intreatment resistance, highlighting the interplay between lactylation andtherapeutic response. We discuss current and emerging strategies that targetlactylation, providing a foundation for future therapeutic development aimed atovercoming resistance and improving cancer treatment outcomes.

**Key points:**

- Lactylation links glycolysis to tumor progression and therapeutic response.
- Modulating lactylation writers and erasers restores treatment sensitivity.
- Aberrant lactylation drives resistance tomultiple cancer therapies.
- Crosstalk with other post‐translational modifications suggests novel combination strategies.

## Graphical abstract

Aberrant lactylation plays a crucial role in the development of resistance to chemotherapy, immunotherapy, targeted therapy, and radiotherapy. Strategies to overcome this resistance include the inhibition of key glycolytic enzymes, targeting lactate transporters, synergistic therapeutic approaches, and the inhibition of specific site.

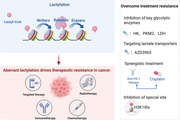

## 1 INTRODUCTION

Under oxygen‐rich conditions, cancer cells preferentially rely on glycolysis rather than the more energy‐efficient oxidative phosphorylation for energy production. This phenomenon is known as the Warburg effect, or aerobic glycolysis.^1^ Under the Warburg effect, pyruvate in tumour cells does not fully enter the mitochondria for oxidative phosphorylation. Instead, lactate dehydrogenase (LDH) catalyzes its reduction to lactate in the cytoplasm.^2^ The connection between lactate and the Warburg effect is a crucial aspect of cancer metabolism. The increased lactate production in cancer cells is associated with various cellular mechanisms, including not only the common hypoxia but also angiogenesis,^3^ macrophage polarization,^4^, ^5^ and T‐cell dysfunction,^6^ as revealed in recent studies. This phenomenon is involved in the metabolic reprogramming of tumour cells and is closely related to the regulation of the tumour microenvironment, cell signalling pathways, as well as tumour invasion and metastasis.^7^, ^8^

**FIGURE 1:**
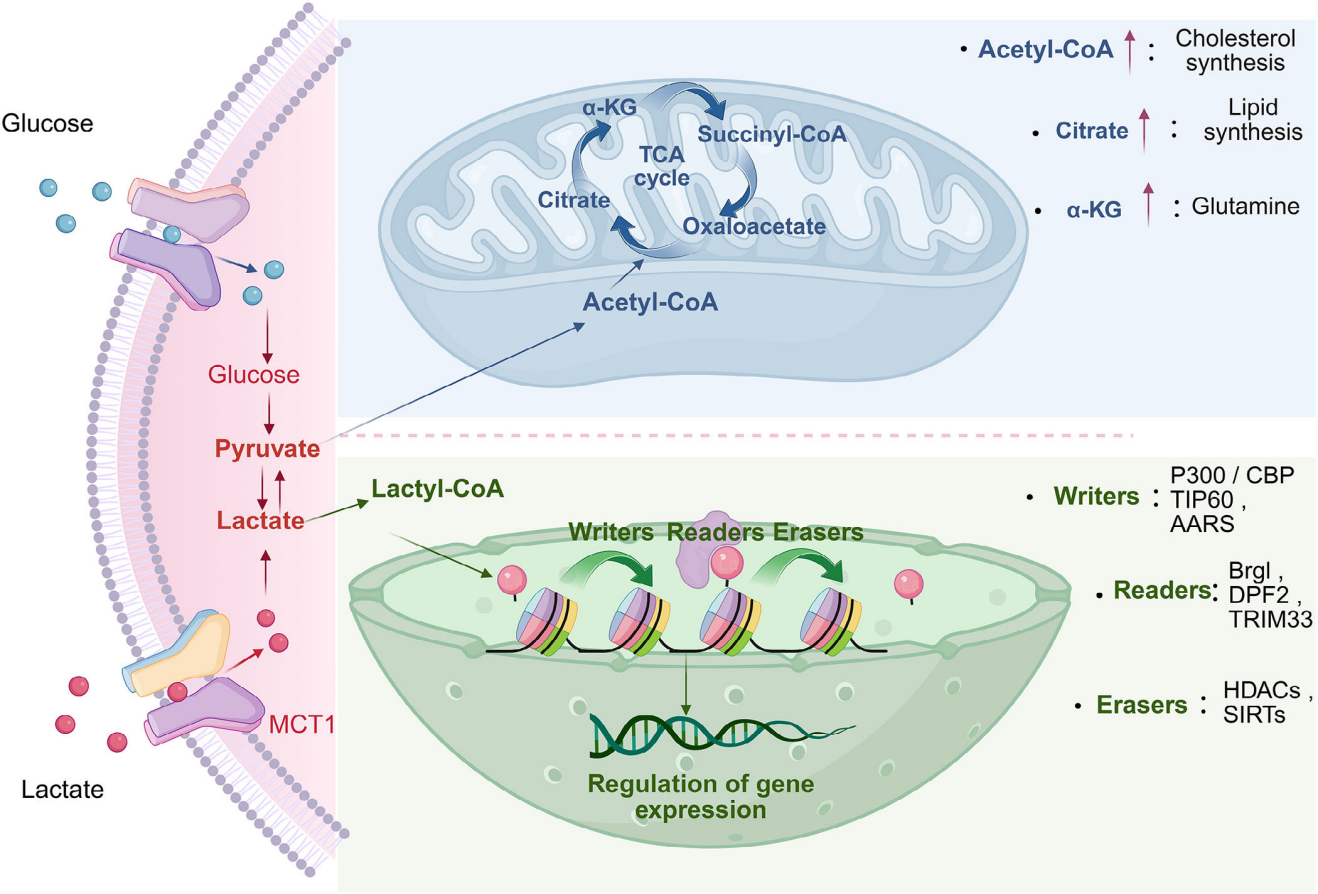
The Warburg effect and lactylation.

In 2019, Zhao et al.^9^ pioneered the discovery of lactate‐driven lysine lactylation as a novel mechanism governing chromatin accessibility and transcriptional control. The discovery of lactylation has expanded the field of epigenetics. According to researchers, lactate is transported across the membrane with the help of monocarboxylate transporters (MCTs), undergoing a series of reactions to convert into acetyl‐CoA, which then participates in the tricarboxylic acid cycle as an energy source.^10^ Intracellular lactate is partially catalyzed to generate lactyl‐CoA. Lactyl‐CoA, the donor molecule required for histone lactylation, is synthesized in an ATP‐dependent manner. The process involves an initial conversion of lactate to a lactyl‐AMP intermediate, followed by its conjugation with coenzyme A to form lactyl‐CoA. To date, two enzymes have been identified as responsible for lactyl‐CoA synthesis: ACSS2 (acyl‐CoA synthetase short‐chain family member 2) and GTPSCS (GTP‐specific succinyl‐CoA synthetase). ACSS2 functions within the nucleus by forming a complex with the histone acetyltransferase KAT2A, thereby facilitating histone lactylation.^11^ In contrast, GTPSCS interacts with the acetyltransferase p300 to form a functional lactyltransferase complex that also catalyzes histone lactylation.^12^ Although ACSS2 and GTPSCS generate lactyl‐CoA through distinct enzymatic mechanisms, both enzymes connect lactate accumulation, from the Warburg effect in tumour cells to histone modifications and gene expression regulation. Thereafter, acyltransferases act as “Writers,” transferring lactyl groups from lactyl‐CoA to the side chains of amino acid residues, recognized and bound by “Readers.” Similarly, this modification can also be reversed by “Erasers”, specifically deacylases, restoring the original state of the amino acid residues^13^ (Figure 1).

Lactylation serves as a critical regulator of cellular metabolism, particularly in the context of metabolic reprogramming. It can directly influence the activity of metabolic enzymes, thus regulating the utilization of energy metabolism pathways.^14^ Lactylation occurs not only on histones but also on non‐histone proteins. As two distinct types of protein modifications, histone lactylation regulates tumour‐related gene transcription, mediating the degradation of tumour suppressors and promoting processes like tumour proliferation, metastasis, invasion, angiogenesis, and immune tolerance. It may also interact with other post‐translational modifications (PTMs). Non‐histone lactylation, on the other hand, influences tumour progression by altering protein structure and function.^9^ In tumour cells, lactylation further modulates tumour progression by regulating gene expression.^15^ Additionally, emerging evidence indicates lactylation might interact with other metabolism‐related modifications, notably acetylation, with both modifications contributing to the development of sepsis.^16^ These findings indicate the potential mechanisms of lactylation in gene expression regulation and its crucial role in tumour cell metabolism and survival. Despite therapeutic innovations, persistent treatment resistance remains an intractable challenge in clinical oncology. Contemporary research have shown that lactylation is associated with tumour drug resistance, and inhibiting lactylation can improve therapeutic outcomes. This review summarizes the mechanisms of lactylation and its regulatory role in modulating resistance to common cancer therapies, including chemotherapy, immunotherapy, targeted therapy, and radiotherapy.

## 2 LACTYLATION IN CHEMORADIOTHERAPY

Malignancies persist as a predominant contributor to the global mortality burden. While surgical intervention remains the cornerstone for localized disease management, its therapeutic limitations in advanced‐stage cancers have established adjuvant chemotherapy as an indispensable component of comprehensive treatment protocols.^17^ Over time, some patients may experience reduced efficacy of chemotherapy, likely due to drug resistance, which can lead to treatment failure and even cancer recurrence. Understanding the mechanisms behind chemotherapy resistance could provide new insights for developing targeted therapies and improving patient survival.

**FIGURE 2:**
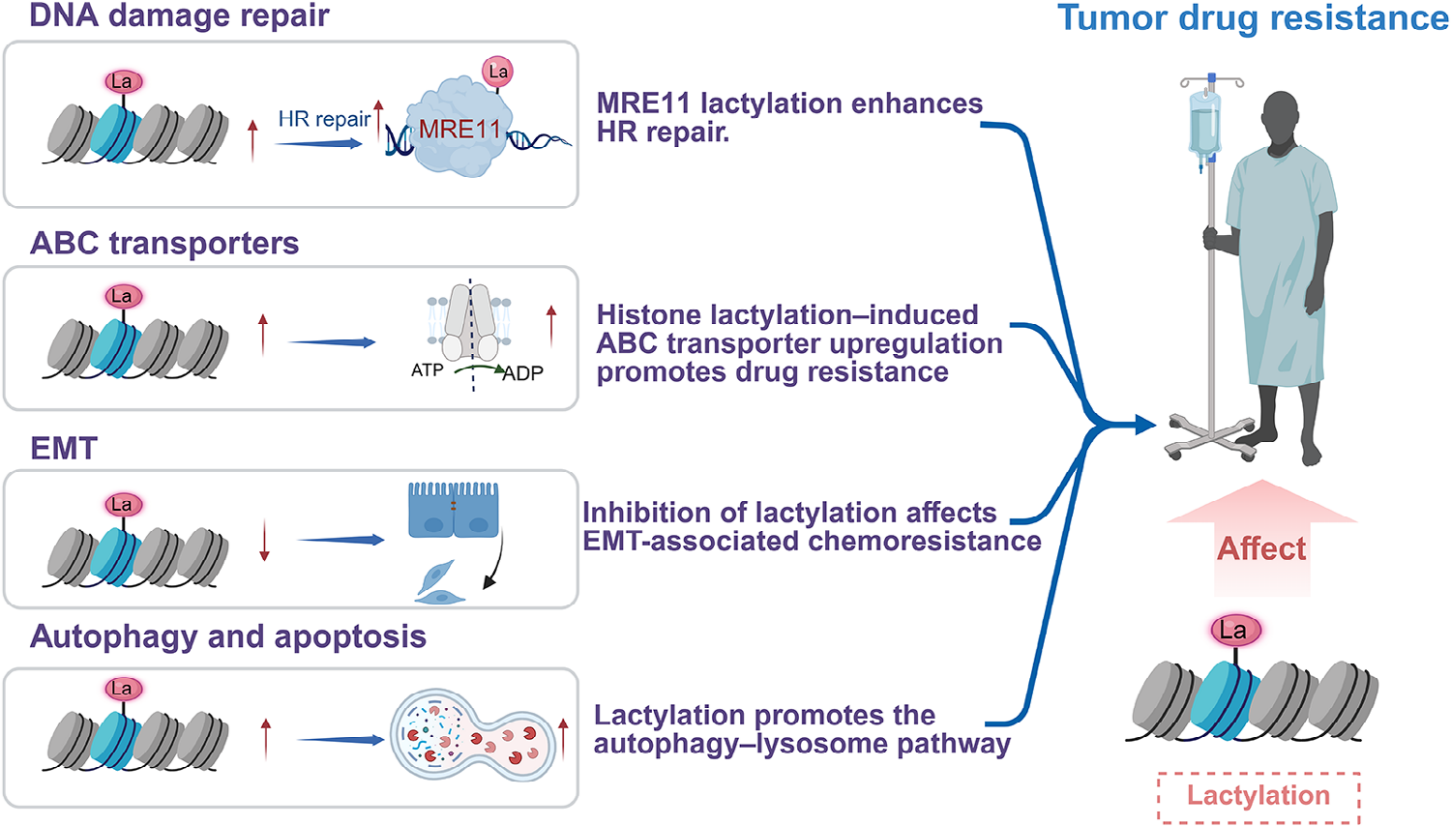
Lactylation and several main drug resistance mechanisms.

Tumour resistance is a complex phenomenon involving multiple mechanisms, which can act independently or in combination to reduce chemotherapy effectiveness. Recent studies have shown that lactylation contributes to chemotherapy resistance through mechanisms such as enhanced DNA damage repair, epithelial–mesenchymal transition (EMT), and overexpression of ATP‐binding cassette (ABC) transporters. Understanding these pathways could inform future drug development (Figure 2).

### 2.1 DNA damage repair

Cisplatin is a widely used chemotherapy drug, particularly effective in the treatment of testicular and ovarian cancers. Combining surgery with cisplatin therapy has been shown to significantly improve patient survival rates.^18^, ^19^ Cisplatin typically forms DNA crosslinks, interfering with DNA replication and transcription, ultimately leading to cell apoptosis.^20^ However, over time, patients may develop resistance, leading to treatment failure.

Recent studies have demonstrated that cancer cells are capable of using lactate to counteract the damage caused by chemotherapy. In a published study, Chen et al.^21^ conducted a comparison between post‐surgical specimens from gastric cancer patients who had undergone platinum‐based neoadjuvant chemotherapy (NAC) and those from patients who received conventional treatments. They found that lactate dehydrogenase (LDHA), responsible for lactate production, was one of the most upregulated proteins in drug‐resistant tumours, and lactate was one of the most abundant metabolites in these tumours. This suggests a strong link between lactate, DNA damage repair, and chemotherapy resistance.

**FIGURE 3:**
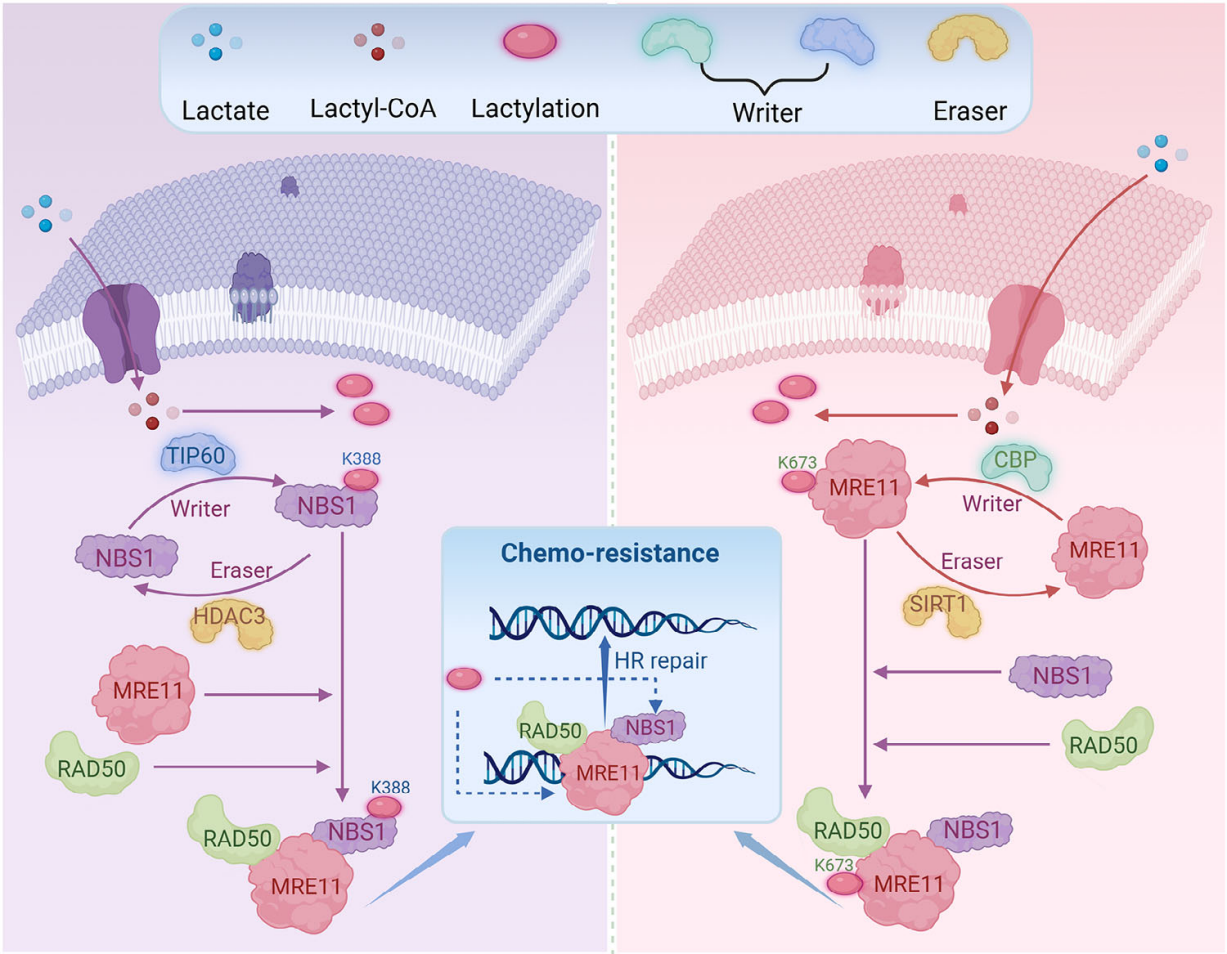
Mechanisms governing lactylation and overcoming resistance to chemoradiotherapy.

Further investigation revealed that lactate significantly enhances homologous recombination repair (HR) in cancer cells by mediating DNA damage repair. The researchers hypothesized that lactylation, a PTM, could be involved. Previous studies have shown that lactylation, as an epigenetic modification, can drive tumour progression and promote immune evasion.^22^ In Chen's study, they identified DNA repair‐related proteins with elevated lysine lactylation levels, particularly NBS1, a key component of the MRN complex (MRE11‐RAD50‐NBS1), which plays a critical role in detecting DNA double‐strand breaks and activating repair pathways. When lysine 388 of NBS1 is lactylated, the interaction between MRE11‐RAD50 and NBS1 is enhanced, promoting HR. Notably, the study also found that stiripentol, an approved anti‐epileptic drug, significantly reduced lactate production and lactylation at the NBS1 Lys388 site (Figure 3). Overall, this research confirms the crucial role of lactylation in tumour survival and is the first to demonstrate that lactylation enhances DNA damage repair in cancer cells, highlighting stiripentol's potential to improve chemotherapy outcomes.

Coincidentally, in the same year, another study published a close relationship between lactylation, DNA damage repair, and chemotherapy resistance in tumour cells.^23^ In this study, researchers screened several key HR proteins and found that MRE11 exhibited the highest levels of lactylation. Lactylation at lysine 673 of MRE11 enhances its binding affinity to DNA, thereby promoting HR. Additionally, the study developed a cell‐penetrating peptide that blocks MRE11 lactylation, which inhibits HR and enhances cancer cell sensitivity to cisplatin (Figure 3).

As early as 2007, Williams et al.^24^ reported the close connection between the MRE11‐RAD50‐NBS1 complex and DNA damage repair, but it was not until recently that lactylation was identified as a key player in bridging chemotherapy resistance and DNA repair. These studies reveal that both NBS1 and MRE11 undergo lactylation and play roles in DNA repair, mediating chemotherapy resistance. The key difference lies in their roles: NBS1 lactylation promotes the formation of the MRN complex and its recruitment to DNA damage sites, while MRE11 lactylation enhances the complex's binding and DNA resection. The role of RAD51, the third component of the MRN complex, remains unclear, potentially offering a new direction for future research.

These findings indicate that lactylation may drive tumour cell adaptation under sustained DNA damage by selectively enhancing HR, contributing to resistance against DNA‐damaging agents. Given HR's critical role in DNA repair during cancer treatment, lactylation may alter tumour cell sensitivity to certain chemotherapies. Understanding this mechanism offers potential avenues for targeting lactylation to overcome chemotherapy resistance.

### 2.2 Overexpression of ABC transporters

Cancer cells frequently exhibit intrinsic or acquired resistance to multiple chemotherapeutic agents, limiting treatment efficacy. One of the main mechanisms of multidrug resistance is the overexpression of ABC transporters, particularly ABCB1, ABCG2, and ABCC1, which are known to contribute to chemotherapy resistance.^25^ Pemetrexed, a rare chemotherapy drug that can effectively cross the blood‐brain barrier, is often limited in treating brain metastases (BM) of lung cancer due to resistance.^26^

In Sun's study,^27^ lactylation plays a critical role in chemotherapy resistance by regulating ABC transporter expression. Upon exposure to agents such as irinotecan, tumour cells adopt a dormancy‐like state characterized by slow proliferation and drug resistance. Downregulation of SMC4 elevates the expression of three glycolytic enzymes, increasing lactate production, which in turn promotes ABC transporter expression via histone lactylation. SMC4 also regulates PGAM1 transcription, and dual loss of SMC4 and PGAM1 disrupts F‐actin assembly, causing cell division failure and polyploidy, ultimately suppressing tumour proliferation. These findings indicate that lactylation mediates drug efflux through ABC transporter regulation, highlighting its potential as a therapeutic target to overcome ABC transporter–driven resistance.

### 2.3 Epithelial–mesenchymal transition

EMT is a persistent challenge in tumour metastasis, with over 90% of malignant tumours originating from epithelial tissues, such as the lung, breast, liver, and gastric cancers. EMT plays a crucial role in the metastasis of these malignancies.^28^ Recent studies have revealed that bladder cancer (BCA) patients frequently develop resistance to platinum‐based chemotherapy, particularly cisplatin, with lactylation playing a key role in driving this resistance.

Using single‐cell RNA sequencing, researchers mapped the single‐cell landscape of BCA, identifying a distinct subpopulation with marked cisplatin resistance. They found that H3K18la was enriched at promoter regions, crucial for activating target gene transcription. Inhibiting H3K18la restored cisplatin sensitivity in resistant epithelial cells. Moreover, YBX1 emerged as a potential target to reverse cisplatin resistance, while YY1 was recognized as a key driver of chemotherapy resistance across various malignancies.^29^ These findings deepen the understanding of the mechanisms underlying cisplatin resistance and offer valuable insights into identifying new intervention targets for overcoming BCA resistance.

### 2.4 Other resistance mechanisms

Autophagy and apoptosis in tumour cells are common mechanisms of drug resistance, with numerous studies demonstrating the involvement of autophagy in chemotherapy resistance across various cancer types.^30^, ^31^, ^32^ In contrast, apoptosis tends to weaken chemotherapy resistance, and pathological mechanisms such as the overexpression of anti‐apoptotic proteins and abnormalities in the DNA damage response have been linked to resistance.^33^, ^34^ Lactate‐induced lysine 91 lactylation of TFEB prevents WWP2‐mediated ubiquitination and proteasomal degradation, enhancing lysosomal activity and autophagic flux. Elevated TFEB lactylation is evident in primary pancreatic ductal adenocarcinoma. Similarly, lactate‐driven Vps34 lactylation increases its lipid kinase activity, promoting autophagy and the endosome–lysosome degradation pathway critical for tumour progression.^35^, ^36^ In the future, lactylation's role in regulating tumour cell autophagy and apoptosis in mediating resistance may present significant challenges for cancer therapy.

As one of the most important metabolites in the tumour microenvironment (TME), lactate is closely associated with tumour immunity. The TME can be divided into cellular and non‐cellular components, with the former enhancing drug resistance by recruiting and secreting various protective cytokines, while the latter mediates resistance by constructing physical barriers and influencing tumour cell growth and metabolism.^37^, ^38^ The complex nature of the TME affects the metabolic state of both tumour and immune cells, and metabolites such as lactate can directly impact tumour resistance.^39^ Understanding lactate metabolism and lactylation in the TME provides key insights into the mechanisms of resistance, which will be further explored in the following sections.

The interaction between acetylation and lactylation is a particularly intriguing area of research. These two modifications share similar enzymatic mechanisms. Traditionally, lysine acetyltransferases (KATs) are considered to catalyze acetylation via acetyl‐CoA. According to existing research, GCN5‐related N‐acetyltransferase, p300‐CBP (CREB‐binding protein), and Sas2 and Tat‐interacting protein 60 (TIP60) are the three most common enzymes involved.^40^ Among them, p300 has also been confirmed to promote histone lactylation in various cellular environments.^41^ In fact, nearly all KATs exhibit some degree of lactylation activity. However, the lactylation efficiency of p300 and other acetyltransferases is relatively low, possibly due to the larger size of the lactyl‐CoA group, which creates spatial hindrance.^11^ Therefore, other mechanisms, such as the lactyl‐CoA‐independent pathway mediated by AARS1/2, also play a complementary role in regulating the lactylation process within the cell.^42^

Furthermore, dual‐specificity enzymes, such as histone deacetylases (HDACs) and sirtuins (SIRTs), also play a central role in regulating both acetylation and lactylation. Lysine deacetylases (KDACs) are divided into two families: Zn2+‐dependent (HDACs, also known as class I, II, and IV KDACs) and NAD+‐dependent SIRTs (also known as class III KDACs).^43^ Studies have shown that enzymes within the KDAC family may also possess the ability to remove lactylation. It has been found that pan‐HDAC inhibitors, such as sodium butyrate and trichostatin A, as well as class I HDAC inhibitors like apicidin, can increase overall histone lactylation.^41^

Research indicates that competition between acetylation and lactylation at the same lysine residue is a key factor in determining the effects of these modifications. For example, specific histone sites, such as H3K9 and H3K18, have been found to undergo both acetylation and lactylation simultaneously.^6^, ^44^ The balance of these modifications may affect gene expression and chromatin dynamics in tumour cells. The interaction between acetylation and lactylation could also contribute to the development of treatment resistance, particularly in cancer therapies targeting epigenetic modifications.

## 3 LACTYLATION IN IMMUNOTHERAPY

Immunotherapy using immune checkpoint inhibitors has been a major breakthrough in oncology over the past decade, demonstrating efficacy across various malignancies. However, like other anti‐cancer treatment modalities, immune checkpoint inhibitors can also encounter resistance issues.^45^ Current research suggests that cancer progression and metastasis involve more than just cancer cells. A disorganized tissue environment, comprising cancer cells, immune cells, and stromal cells—known as the TME—drives cancer's progression and spread.^46^ The TME sustains and promotes tumour growth, with each component potentially playing a critical role in cancer development. Studies have shown that targeting elements within the TME may significantly help in inhibiting tumour progression.^47^ Thus, understanding tumour immunity within the tumour immune microenvironment is essential for designing and optimizing immunotherapeutic strategies.

**FIGURE 4:**
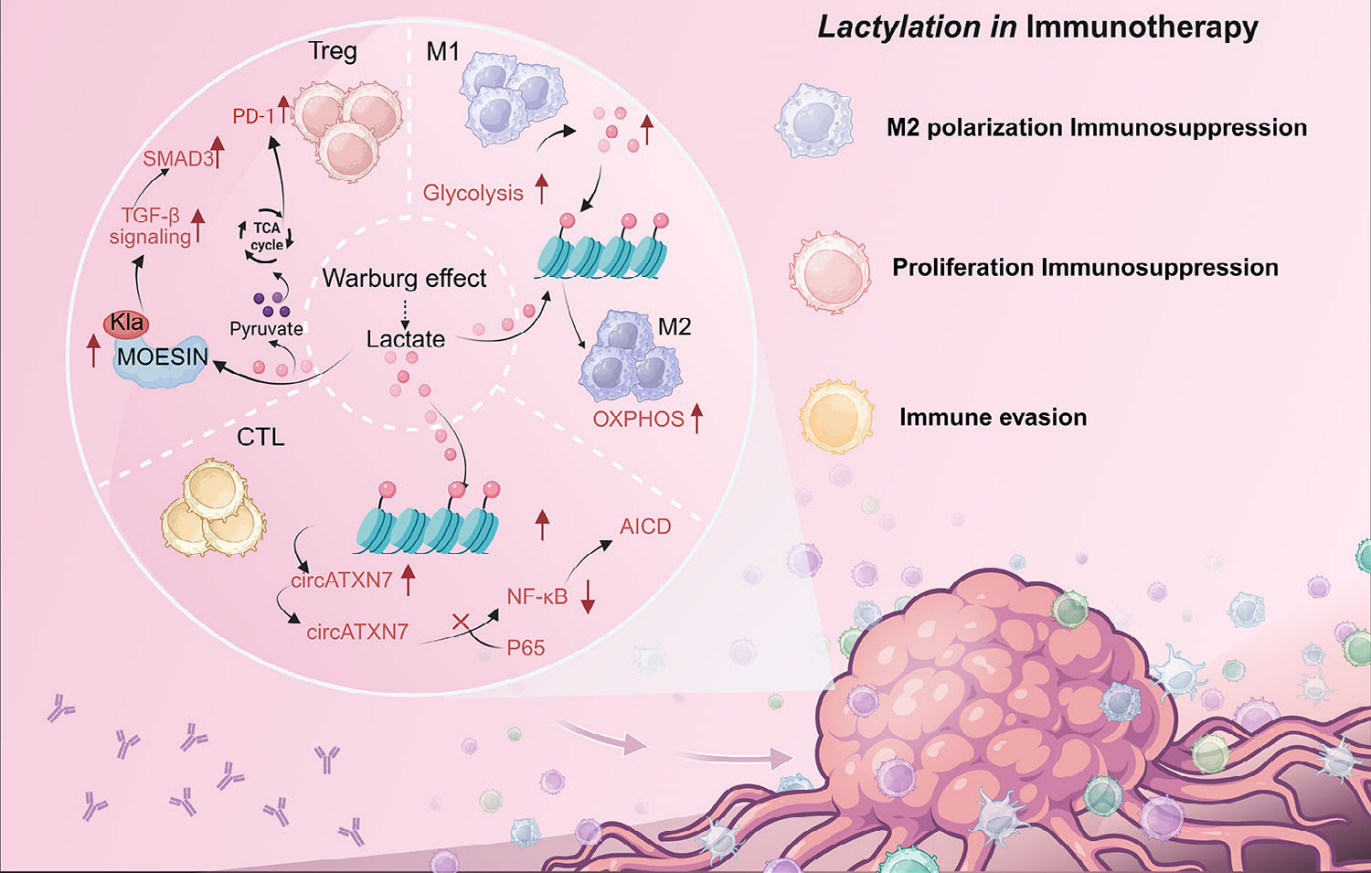
Lactylation‐mediated immunosuppression and immune escape.

The current enthusiasm for cancer immunotherapy largely stems from the success of monoclonal antibodies targeting immune suppression within the TME. These antibodies block the immune inhibitory receptor programmed death 1 (PD‐1) and its ligand B7‐H1, also known as programmed death ligand 1 (PD‐L1).^48^ Therapies targeting the PD‐1/B7‐H1 axis, known as anti‐PD therapies, have shown durable therapeutic effects across various cancer types.^49^, ^50^ In cancer, immune evasion and metabolic reprogramming are two fundamental hallmarks, and lactate tightly links these processes. Excess lactate helps create an immunosuppressive environment that favours cancer growth and plays a crucial role in shaping immune cell function.^51^ Elevated lactate levels are also closely associated with poor prognosis in cancer patients, and both in vivo and in vitro studies have shown that adding exogenous lactate promotes cancer progression and resistance.^52^ Previous studies have demonstrated that lactate and lactylation can upregulate PD‐L1 expression in macrophages, thereby mediating immune resistance. More recent research has revealed that lactylation in immune‐related cells within the TME, such as tumour‐associated macrophages (TAMs), regulatory T cells (Tregs), and cytotoxic T lymphocytes (CTLs), directly or indirectly promotes tumour immune suppression and immune evasion, leading to resistance to immunotherapy (Figure 4).

### 3.1 M2 polarization immunosuppression

Tumour‐associated macrophages are a key component of tumour tissues and organs, consisting primarily of M1 macrophages, which hinder cancer cell proliferation, and M2 macrophages, which promote functional differentiation. In Chaudagar's study,^53^ tumour cells, through the Warburg effect, increased lactate production, which accumulated in the TME and promoted the polarization of M2‐type TAMs, leading to immune suppression. Lactylation was found to affect macrophage function via the PD‐1 pathway, contributing to this immunosuppressive state.^54^ Furthermore, lactate enhances lysine 62 lactylation on pyruvate kinase M1/2 (PKM), increasing its enzymatic activity, inhibiting the Warburg effect, and ultimately promoting the shift from M1 to M2 TAMs. M2‐type TAMs are formed by absorbing lactate produced by tumour cells, with lactate further stimulating the expression of vascular endothelial growth factor (VEGF) and HIF‐1α.^55^, ^56^ These findings suggest that elevated lactylation levels are associated with the immunosuppressive state of TAMs, allowing tumour cells to evade immune surveillance and increasing resistance to immunotherapy.

In addition to immune suppression caused by M2 macrophage polarization, other macrophages in the innate immune system can regulate lactylation and suppress innate immunity, contributing to immunotherapy resistance. Zong's study^57^ identified AARS1 as a lactate sensor and lactate transferase that mediates lactylation in tumours, targeting the tumour suppressor gene P53 and promoting tumorigenesis. Li's research^58^ further showed that AARS1 and AARS2 act as lactate sensors, directly binding L‐lactate and catalyzing lactylation without relying on coenzyme A. Under high‐lactate conditions, AARS2 interacts with cGAS, mediating lactylation at key lysine residues (Lys131 in humans, Lys156 in mice), which inhibits cGAS's ability to bind DNA and produce cGAMP, thereby suppressing the innate immune response and facilitating immune evasion. These studies reveal the essential role of AARS1/2 in lactate sensing and lactylation, shedding light on how lactate accumulation and lactylation contribute to cancer and providing new perspectives on immunotherapy resistance.

Additionally, in De's study,^22^ in glioblastoma (GBM), PERK‐driven glucose metabolism enhances the immunosuppressive activity of monocyte‐derived macrophages (MDMs) via histone lactylation. Targeting this pathway may improve immunotherapy efficacy, offering a potential GBM treatment strategy. Additionally, tumour microenvironment lactate induces METTL3 upregulation in myeloid‐derived macrophages through histone lactylation, which drives the immunosuppressive function of tumour‐infiltrating myeloid cells.^59^ Therefore, targeting METTL3 inhibition could reduce the immunosuppressive function of tumour‐infiltrating myeloid cells, potentially enhancing resistance to cancer therapies.

### 3.2 Proliferation immunosuppression

Tregs, as adaptive immune cells, are key immunoregulatory components within the TME, with their proliferation, function, and metabolic adaptation all dependent on TGF‐β signalling.^60^, ^61^, ^62^ Interestingly, lactate can activate transforming growth factor‐β (TGF‐β) signalling in various cell types.^63^, ^64^ In Treg cells, TGF‐β signalling is activated through lactylation of Lys72 in MOESIN, enhancing the interaction between MOESIN and TGF‐β, thereby promoting downstream signalling.^65^

Chen's study^66^ revealed that lactate enhances lactylation at the Lys70 site of apolipoprotein C‐II (APOC2), stabilizing it and leading to free fatty acid release, Treg‐cell accumulation, immunotherapy resistance, and metastasis. The researchers developed an anti‐APOC2 K70‐lac antibody, which enhanced sensitivity to anti‐PD‐1 therapy in vivo. This discovery highlights the potential of targeting lactyl‐APOC2‐K70 as a novel combination therapy to improve immunotherapy responses. Additionally, other studies^67^ found that lactate promotes Lys72 lactylation of MOESIN, impairing its function, facilitating Treg cell generation, and enabling tumour immune evasion. Inhibiting MOESIN lactylation may enhance immunotherapy efficacy by limiting Treg‐mediated suppression. Similarly, lactate from patient‐derived glioma stem cells and microglia/macrophages drives histone lactylation–mediated epigenetic reprogramming in GBM cells, inducing an immunosuppressive transcriptional program and upregulating the “don't eat me” signal CD47 to inhibit phagocytosis. Mechanistically, lactylated histones interact with CBX3, which, despite lacking lactyltransferase activity, binds histone acetyltransferase EP300, enhancing its specificity for lactyl‐CoA and promoting an immunosuppressive cytokine profile.^68^

### 3.3 Immune evasion

As another key player in adaptive immunity, CTLs are undoubtedly essential for anti‐tumour and anti‐infection responses. PD‐(L)1 inhibitors, CAR‐T therapy, and CD3 T‐cell bispecific antibodies are all successful applications of CTL cytotoxicity.^69^ In Zhang's study,^70^ it was found that lactate‐driven H3K18la promotes tumour progression. Blocking H3K18la enhanced CD8+ T‐cell‐based anti‐tumour immunity and inhibited immune evasion in non‐small‐cell lung cancer (NSCLC). This immune evasion was driven by POM121, which upregulated PD‐L1 expression by promoting MYC nuclear translocation. Using glycolysis inhibitors to block this pathway can significantly boost CD8+ T‐cell responses and improve the efficacy of anti‐PD‐1 therapy.

Similarly, Wang's study^6^ found that increased histone lactylation is linked to poor immunotherapy response in head and neck squamous cell carcinoma (HNSCC). The specific lactylation site, H3K9la, was identified, with interleukin‐11 (IL‐11) being a downstream regulatory gene. IL‐11 activated immune checkpoint genes through the JAK2/STAT3 pathway in CD8+ T cells, and its overexpression promoted tumour progression and CD8+ T cell dysfunction. This highlights the crucial role of lactylation in immune evasion and offers new insights for immunotherapy strategies in HNSCC.

Additionally, mutant KRAS (KRASMUT) has been shown to induce immune evasion through activation‐induced cell death (AICD). In Zhou's study,^71^ lactate from KRASMUT tumour cells promotes histone lactylation, activating circATXN7, which binds the NF‐κB p65 subunit, masks its nuclear localization signal, and sequesters it in the cytoplasm, thereby inhibiting NF‐κB signalling and increasing CTL sensitivity to AICD. Clinically, circATXN7 expression in KRASMUT‐specific CTLs correlates with poor prognosis and immunotherapy resistance, while its knockout enhances anti‐PD‐1 efficacy. Similarly, during viral infection, virus‐induced lactate facilitates immune evasion by directly inhibiting immune signalling pathways.^72^

Given that lactate accumulation is a hallmark of the tumour microenvironment, the discovery of lactylation has opened new avenues for exploring tumour progression and immune evasion in cancer immunotherapy. While lactylation plays a key role in immune suppression within the tumour microenvironment, its precise mechanisms and implications for immunotherapy resistance remain under investigation. Evidence links lactate accumulation to immune cell dysfunction, but conclusions vary regarding the specific pathways and targets involved. One line of research indicates that lactate can drive M2‐type TAM polarization via the Warburg effect, thereby promoting immune suppression. Another body of work has identified aminoacyl‐tRNA synthetase 1 (AARS1) as a lactate sensor that mediates lactylation to promote immune evasion by targeting tumour suppressor genes such as p53. The former emphasizes metabolic shifts in macrophages, whereas the latter focuses on molecular lactylation, pointing to two distinct therapeutic approaches—targeting metabolic pathways versus the lactylation machinery. Further studies have shown that AARS1 and AARS2 can catalyze lactylation independently of coenzyme A, inhibiting cGAS and suppressing innate immunity to facilitate immune evasion. In contrast, other investigations have demonstrated that lactate‐induced histone lactylation in glioblastoma enhances immunosuppressive activity and that blocking PERK‐driven lactylation may improve immunotherapy outcomes. The former line of evidence centres on the effects of lactylation on innate immunity and tumour progression, whereas the latter highlights its role in epigenetic regulation of macrophage function, offering a broader strategy for targeting histone modifications in immunotherapy.

Across immune cell types, lactylation acts as a metabolic–epigenetic bridge, translating elevated lactate into durable immune suppression. This suppression is mechanistically redundant: TAMs, Tregs, and CTLs are targeted through parallel but converging routes, ensuring immune evasion even if one pathway is blocked. This redundancy may explain the limited efficacy of monotherapies targeting single checkpoints, such as single‐agent PD‐1 blockade, and suggests that combinational approaches inhibiting lactylation alongside ICIs could disrupt multiple resistance nodes simultaneously.

## 4 LACTYLATION IN TARGETED THERAPY

Targeted cancer therapy is a precise approach that focuses on specific molecules within cancer cells to inhibit tumour growth and spread while minimizing harm to normal cells. It offers high specificity, effective tumour inhibition, and fewer side effects compared with chemotherapy and immunotherapy.^73^, ^74^

Targeting lactylation is closely associated with aerobic glycolysis, as inhibiting tumour cell glycolysis presents a promising strategy to reduce lactylation and overcome therapy resistance. Key enzymes like HK2,^75^, ^76^, ^77^, ^78^, ^79^ PKM,^80^, ^81^, ^82^ and LDH,^83^, ^84^, ^85^, ^86^ along with transporters, such as MCT1 and MCT4,^82^, ^87^, ^88^, ^89^ are primary targets in this process.

LDH, a tetrameric enzyme, plays a crucial role in mediating the bidirectional conversion between pyruvate and lactate.^90^, ^91^, ^92^ This metabolic reprogramming diverts the metabolic precursors of pyruvate into the pentose phosphate pathway, thereby providing essential support for cancer cell proliferation.^93^, ^94^ Clinically, elevated levels of LDHA have been consistently associated with poor prognosis in numerous human malignancies.^95^, ^96^

Strong evidence suggests that the combination of MCT‐targeted therapies with other treatment methods can achieve better therapeutic outcomes. Li et al.^97^ demonstrated that, in a breast cancer mouse model, the MCT inhibitor Syrosingopine led to a decrease in the number of regulatory T (Treg) cells and an increase in the number of NK cells, as well as the M1 phenotype of TAM. This finding suggests a reversal of the immunosuppressive tumour microenvironment. Concurrently, Ma et al.^98^ discovered that lithium carbonate (LC) more accurately facilitated the localization of MCT1 to the mitochondrial membrane, increasing the lactate concentration within the mitochondria. The above results led to the revitalization of tumour‐reactive CD8+ T cells, which enhanced the sensitivity of immunotherapy against colorectal cancer (CRC), melanoma, and breast cancer. These findings provide valuable insights into the potential of MCT‐targeted therapy to act in synergy with immunotherapy.

Current drug development targeting lactylation focuses on MCT1 inhibitors^99^ and LDH inhibitors,^66^ while direct targeting of lactylation mechanisms is under exploration. A recent study identified givinostat as an inhibitor of H3K18 lactylation, showing potential as a direct intervention.^100^ One promising compound, dimethyl zelladan, disrupts histone lactylation associated with metabolic stress, demonstrating potential in inhibiting liver cancer stem cells. In Pan's research,^101^ triterpenoid demethylzellal effectively suppressed HCC by inhibiting H3 histone lactylation, enhancing the efficacy of anti‐cancer therapies (Table 1).

**TABLE 1:** The potential attempts of targeting lactylation in cancer therapy.

Targets	Drugs	Cancer	Reference
HK	2‐DG,	Ocular melanoma,	^75^
	3‐BP,	hepatocellular carcinoma,	^76^
	Limonin,	hepatocellular carcinoma,	^77^
	Lonidamine	melanoma and breast cancer	^78^, ^79^
LDH	Oxamate,	Ocular melanoma,	^83^
		gastric carcinoma,	^84^
	Gossypol,	melanoma,	^85^
	PS^TM^B	colon cancer	^86^
PKM	Compound 3K,	Ovarian cancer,	^80^
	Shikonin,	bladder cancer,	^81^
		hepatocellular carcinoma	^82^
MCTs	CHC,	Colorectal cancer,	^82^
	DIDS,	prostate cancer,	^87^
	Simvastatin,	oral squamous cell carcinoma,	^88^
	Quercetin	breast cancer	^89^

**FIGURE 5:**
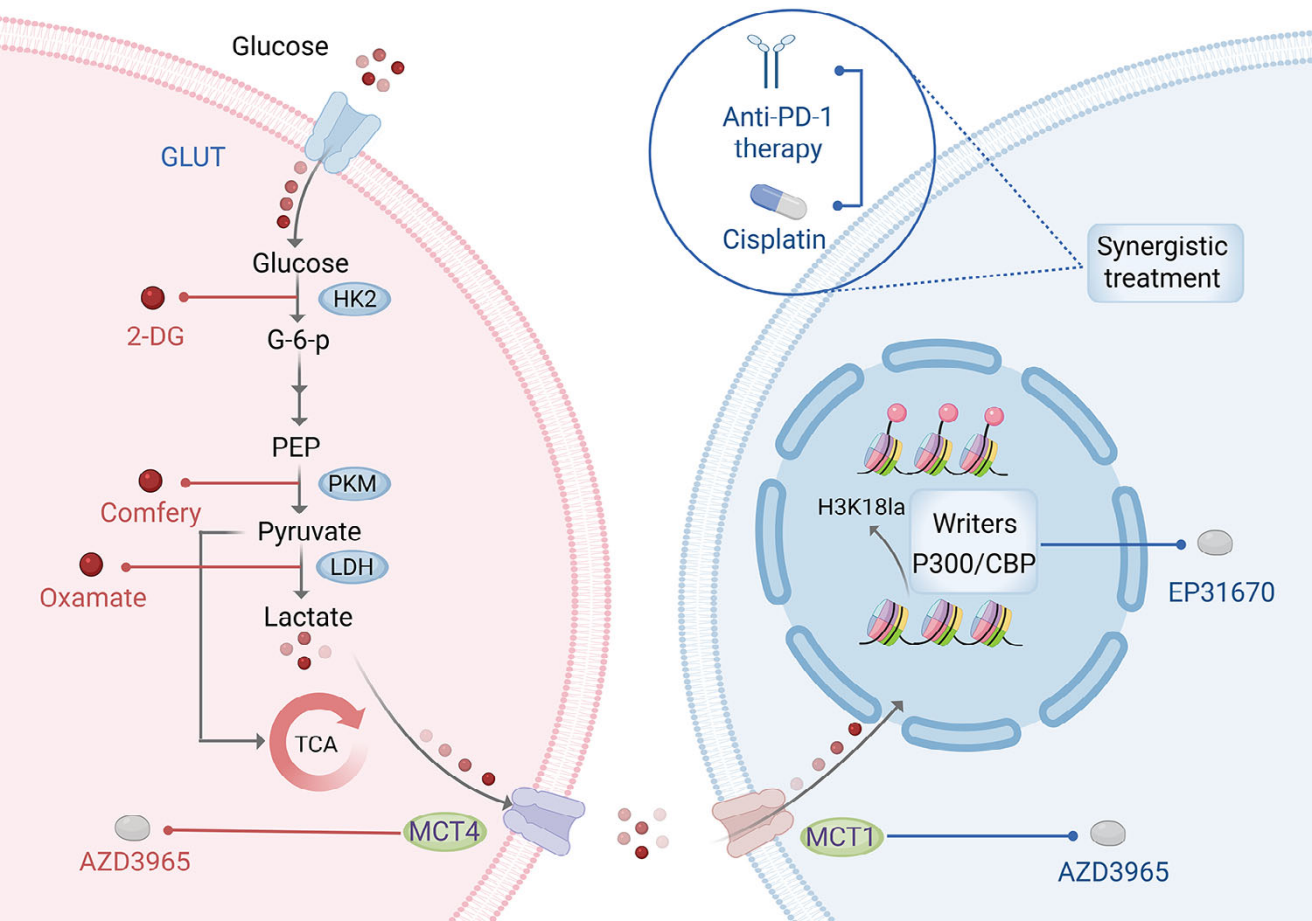
Targeting lactate production and lactylation to enhance anticancer therapy.

Bevacizumab, an anti‐angiogenic therapy targeting VEGFA, plays a crucial role in treating advanced CRC by blocking VEGFA‐VEGFR binding to inhibit angiogenesis.^102^ While it improves outcomes in metastatic CRC, resistance often develops. Li's study^103^ identified histone lactylation as a critical mechanism of bevacizumab resistance in CRC. In resistant patients, elevated histone lactylation upregulates RUBCNL/Pacer transcription, promoting autophagosome maturation via BECN1 interaction and supporting tumour survival under hypoxia. Inhibiting histone lactylation suppresses tumour growth in hypoxic CRC, and combined inhibition of lactylation and autophagy with bevacizumab markedly improves therapeutic efficacy. This highlights lactylation's essential role in hypoxia‐adapted tumour survival and offers a strategy to overcome bevacizumab resistance (Figure 5).

Across targeted therapies, lactylation functions as a metabolic–epigenetic integrator that enables tumours to bypass inhibition of their primary oncogenic pathway. Whether driven by glycolytic flux, hypoxia, or transporter‐mediated lactate exchange, the outcome is consistent: stabilization of pro‐survival transcriptional programs and maintenance of a resistant tumour phenotype. This redundancy explains why both metabolic interventions—such as LDH or MCT blockade—and direct epigenetic strategies employing lactylation inhibitors demonstrate cross‐therapy potential. Future targeted therapy design should integrate lactylation suppression as a co‐strategy to prevent the metabolic plasticity that underlies convergent resistance mechanisms.

## 5 LACTYLATION IN RADIOTHERAPY

Radiation therapy (RT) is widely known for inducing DNA damage through ionizing radiation (IR), either by directly causing DNA breaks or by generating highly reactive free radicals through the absorption of high‐energy wavelengths, which indirectly damage DNA.^104^ RT leads to single‐strand breaks, double‐strand breaks, and ablated DNA sites in tumour cells, prompting the cells to activate a series of complex protective and repair mechanisms to survive.^105^ DNA damage sensors trigger cell cycle arrest and initiate a cascade of responses to activate downstream effectors. Common examples include mediator of DNA damage checkpoint protein 1 (MDC1) and tumour suppressor p53‐binding protein 1 (53BP1), which detect DNA damage and activate downstream pathways.^106^, ^107^ Once activated, kinases amplify the damage signals, making regulation of DNA repair mechanisms critical in modulating tumour radiosensitivity.^108^

Recent pivotal studies have demonstrated that lactate enhances tumour resistance to RT by promoting DNA damage repair.^21^, ^23^ Additionally, lactylation has been implicated in GBM radioresistance. High expression of aldehyde dehydrogenase 1 family member A3 (ALDH1A3) is linked to poor prognosis in GBM and drives enhanced glycolysis, contributing to RT resistance. In this context, lactate accumulation mediates lactylation at the Lys247 site of XRCC1, a DNA repair protein, promoting DNA damage repair in GBM. Researchers identified a small molecule, D34‐919, that blocks the interaction between ALDH1A3 and PKM2, restoring tumour cell sensitivity to radiotherapy.^109^

These findings reveal that RT induces DNA repair enhancement, and lactate/lactylation strengthens tumour resistance to RT, reducing treatment efficacy. Understanding these mechanisms offers potential avenues to improve radiosensitivity in future cancer therapies.

## 6 CLINICAL TARGETING OF LACTYLATION AND LACTATE METABOLISM IN CANCER

Lactate metabolism has become a crucial target for cancer therapy, with its dysregulation serving as a fundamental mechanism driving tumour progression and metastasis. Lactate production and transport are primarily mediated by LDH and MCTs, with LDH catalyzing the conversion of pyruvate to lactate and MCTs facilitating lactate efflux, contributing to the acidic tumour microenvironment that promotes immune evasion. Targeting LDH and MCTs has therefore become a promising strategy for disrupting tumour metabolism and improving cancer therapy. Inhibition of LDH, particularly the LDHA isoform, has been shown to reduce lactate production, thereby reprogramming tumour metabolism and inhibiting cancer cell proliferation.^110^ Similarly, targeting MCTs has been investigated for its potential to block lactate transport and thereby reduce the lactate‐induced acidic microenvironment.^111^ Recent clinical trials have focused on evaluating the efficacy of LDH inhibitors, such as AT‐101 (gossypol),^112^, ^113^ and MCT inhibitors like AZD3965,^114^, ^115^ demonstrating their ability to slow tumour progression and enhance the effects of other therapies, such as immunotherapy and chemotherapy.

Advances in detection technologies, such as fluorescent lactate analogues and isotope‐tracing‐based metabolic flux analysis, have significantly enhanced our understanding of lactate's role in tumour biology.^116^ These techniques allow for real‐time tracking of lactate metabolism at the cellular and subcellular levels,^117^ providing valuable insights into how lactate affects various cellular processes, including gene expression, cell cycle progression, and immune cell activation.^118^ For example, single‐cell metabolomics has revealed that lactate metabolism influences the metastatic potential of CRC cells by modulating specific metabolic pathways, such as those associated with the Warburg effect.^119^

The use of lactate‐modifying agents has also been integrated with other therapeutic modalities to improve treatment outcomes. LDH inhibitors, when combined with immune checkpoint inhibitors, have shown potential in enhancing T‐cell infiltration into tumours and reversing the immunosuppressive TME.^110^ This combination approach may overcome the resistance mechanisms that are often observed in tumours that rely heavily on lactate metabolism. Furthermore, the combination of LDH inhibition with photothermal therapy and radiotherapy has been shown to enhance tumour response, making these therapies more effective in treating cancer.^120^ Currently, several ongoing clinical trials are investigating the therapeutic potential of targeting lactate metabolism in various cancer types. For instance, phase I/II trials of AT‐101 in prostate cancer and lymphoma have demonstrated promising results in terms of improving progression‐free survival.^112^, ^113^ Similarly, the MCT1/2 inhibitor AZD3965 has completed early‐phase trials in patients with solid tumours and lymphoma, showing favourable pharmacokinetic properties and tolerability.^114^, ^115^ Additionally, studies exploring the combination of lactate‐targeting agents with immunotherapies, such as PD‐1/PD‐L1 inhibitors, are underway, with encouraging preclinical evidence suggesting enhanced anti‐tumour responses.^121^, ^122^, ^123^ Recent studies have revealed that non‐histone lactylation of ABCF1 plays a significant role in the growth and metastasis of hepatocellular carcinoma (HCC). It has been demonstrated that ABCF1 undergoes lactylation within the nucleus and functions in a transcription factor–like manner. The authors identified and clinically validated the small‐molecule compound Tubuloside A as a potential therapeutic agent targeting ABCF1‐K430la for the treatment of HCC tumours^124^ (Table 2).

**TABLE 2:** Key ongoing clinical trials targeting lactate metabolism and lactylation.

Targets	Inhibitor	Clinical trial phase	Cancer types	Outcome	Reference
LDH	AT‐101 (Gossypol)	Phase I/II	Prostate cancer, lymphoma	Prolonged progression‐free survival	^112^, ^113^
MCT1/2	AZD3965	Phase I/II	Solid tumours, lymphoma	Positive pharmacokinetics, patient tolerability	^114^, ^115^
LDH + Immunotherapy	AT‐101 + Pembrolizumab	Preclinical	NSCLC, melanoma	Enhanced T‐cell infiltration, improved immune response	^122^, ^123^
MCT1 + Immunotherapy	Syrosingopine	Preclinical	Breast cancer	Reversal of immunosuppressive TME, improved therapy sensitivity	^124^
Lactylation	Demethylzellal	Preclinical	Liver cancer	Disrupting metabolic stress‐related histone lactylation	^101^
Lactylation	Tubuloside A	Preclinical	Liver cancer	Targeting the lactylation of ABCF1 in the nucleus	^125^

The complexity of lactate metabolism across different tumour types remains a significant challenge. Tumour cells exhibit metabolic heterogeneity, with variations in lactate production and utilization across different stages of tumour progression and among distinct cancer types. This heterogeneity necessitates the development of more specific lactate‐targeting strategies to avoid potential off‐target effects on healthy cells. Researchers are currently exploring new avenues, such as the use of lactate oxidase (LOx) and PROTAC (proteolysis‐targeting chimaeras), which offer the ability to degrade lactate‐modifying enzymes and modulate lactate levels within tumours.^125^ These novel approaches hold promise for more precise and effective therapeutic interventions. Although numerous studies using cell lines and mouse models have demonstrated the role of lactylation in various cancer‐related processes, many mechanisms have yet to be validated in clinical cohorts or trials. The translational relevance of these findings—particularly in the context of lactylation‐targeted therapeutic interventions—remains to be firmly established. The therapeutic potential of lactylation inhibitors, as well as the broader application of lactylation‐related mechanisms, requires further substantiation in human clinical settings. Therefore, future clinical studies will be essential to determine the efficacy and safety of lactylation‐targeted therapies and to evaluate how these strategies can be integrated with existing treatment regimens for cancer and other diseases.

## 7 CONCLUSION

Since the groundbreaking discovery of lysine lactylation by Professor Yingming Zhao's team at the University of Chicago in 2019, utilizing LC/MS technology,^9^ research studies on lactylation have rapidly advanced. Lactylation has been confirmed across various cell types and is recognized for its pro‐tumour role in cancer. Lactylation, as a form of post‐translational modification, has garnered increasing attention in recent years due to its potential therapeutic significance in various diseases, particularly cancers and metabolic disorders. The regulation of lactylation is mediated by “writers” and “erasers”. Similar to other post‐translational modifications, inhibiting these enzymes is considered a promising therapeutic strategy to modulate lactylation levels and their associated biological effects. Studies on lactylation writer inhibitors have shown encouraging potential. These inhibitors target enzymes responsible for transferring lactyl groups to lysine residues on proteins. For example, p300/CBP inhibitors like A485 have been demonstrated to reduce lactylation and reverse tumour progression in certain cancer models.^42^ On the other hand, lactylation eraser inhibitors, including HDACs and SIRTs, are also regarded as potential therapeutic tools. Inhibition of these enzymes can prevent the removal of lactylation marks, thereby sustaining their regulatory effects on protein function. For instance, the HDAC1 inhibitor TSA has been shown to increase levels of H4K12la.^126^

As numerous studies have demonstrated, regulating lactylation levels can overcome resistance to conventional chemotherapy, immunotherapy, targeted therapy, and radiation therapy. These findings suggest the potential of developing new therapies by reducing lactylation levels to combat cancer resistance. This review has summarized the mechanisms of lactylation and its role in drug resistance; however, there is still a long way to go before clinical application.

First, it remains unclear whether lactylation‐induced resistance is limited to cancers with specific characteristics or applies broadly across various cancers. A deeper understanding of the mechanisms linking lactylation to resistance is essential to define effective treatment strategies. Second, lactylation competes with acetylation, creating a complex balance in the system. The intricate interplay between lactylation and other PTMs represents a promising avenue for future research. Modifications such as acetylation, methylation, and phosphorylation frequently coexist and interact within the same protein, forming a dynamic and context‐dependent regulatory network. Crosstalk between lactylation and these modifications can influence protein function, stability, and intermolecular interactions, ultimately impacting cellular processes including metabolism, immune responses, and cancer progression. Elucidating these interactions may facilitate the identification of novel therapeutic targets and the development of combination treatment strategies capable of modulating multiple PTMs simultaneously, thereby providing a more comprehensive approach to overcoming cancer resistance and other diseases.

## AUTHOR CONTRIBUTIONS

Yanwei Lu and Haibo Zhang designed the study and approved the final submission. Keke Xu, Yiyi Shou, and Ruiqi Liu drafted the manuscript. Hao Xiong, Xiaomeng Dai, Xuanwen Bao, Xiaoyan Chen, Luanluan Huang, and Hailong Sheng assisted in the revision and drafting process.

## CONFLICT OF INTEREST STATEMENT

The authors declare no conflict of interest.

## ETHICS STATEMENT

Ethical approval is not applicable to this study.

## Data Availability

Data sharing is not applicable to this article as no new data were created or analyzed in this study.
